# Author Correction: Brain aging is faithfully modelled in organotypic brain slices and accelerated by prions

**DOI:** 10.1038/s42003-022-03572-w

**Published:** 2022-06-20

**Authors:** Yingjun Liu, Assunta Senatore, Silvia Sorce, Mario Nuvolone, Jingjing Guo, Zeynep H. Gümüş, Adriano Aguzzi

**Affiliations:** 1 Institute of Neuropathology, University of Zurich, Zurich, Switzerland; 2 Amyloidosis Research and Treatment Center, Foundation IRCCS Policlinico San Matteo, Department of Molecular Medicine, University of Pavia, Pavia, Italy; 3 Department of Genetics and Genomics, Icahn School of Medicine at Mount Sinai, New York, NY USA; 4 Precision Immunology Institute, Icahn School of Medicine at Mount Sinai, New York, NY USA; 5 Icahn Institute for Data Science and Genomic Technology, Icahn School of Medicine at Mount Sinai, New York, NY USA

**Keywords:** Neural ageing, Prion diseases

Correction to: *Communications Biology* 10.1038/s42003-022-03496-5, published online 08 June 2022.

In this article the grant number 179040 relating to Swiss National Science Foundation was omitted. The original article has been corrected.

