# Supplementary material for: Brain aging is faithfully modelled in organotypic brain slices and accelerated by prions
Source: Commun Biol. 2022 Jun 8;5:557. doi: 10.1038/s42003-022-03496-5 (PMC9177860; doi:10.1038/s42003-022-03496-5)
Supplement: Supplementary file 1 — Supplementary Information [file 42003_2022_3496_MOESM1_ESM.pdf]

Supplementary fig. 1, Liu et al.

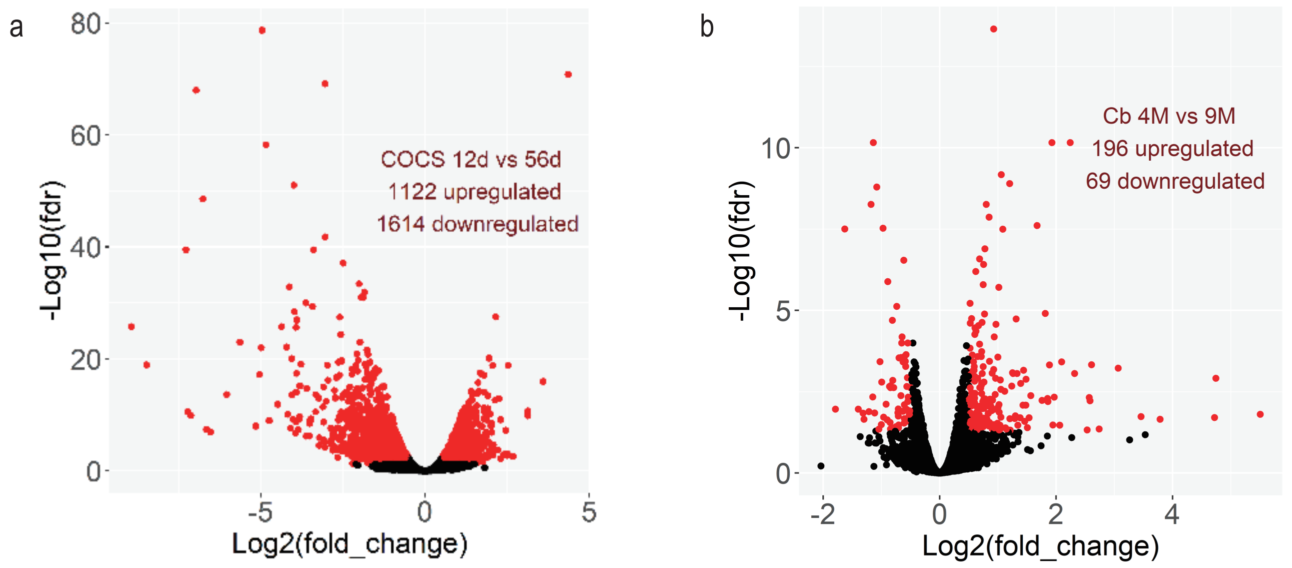

Supplementary figure 1, a, Volcano plot showing the dysregulated genes between the 12-day-old and 56-day-old control COCS. b, Volcano plot showing the dysregulated genes between the 4-month-old (4M) and 9-month-old (9M) NBH mouse cerebellum (Cb).

Supplementary fig 2, Liu et al.

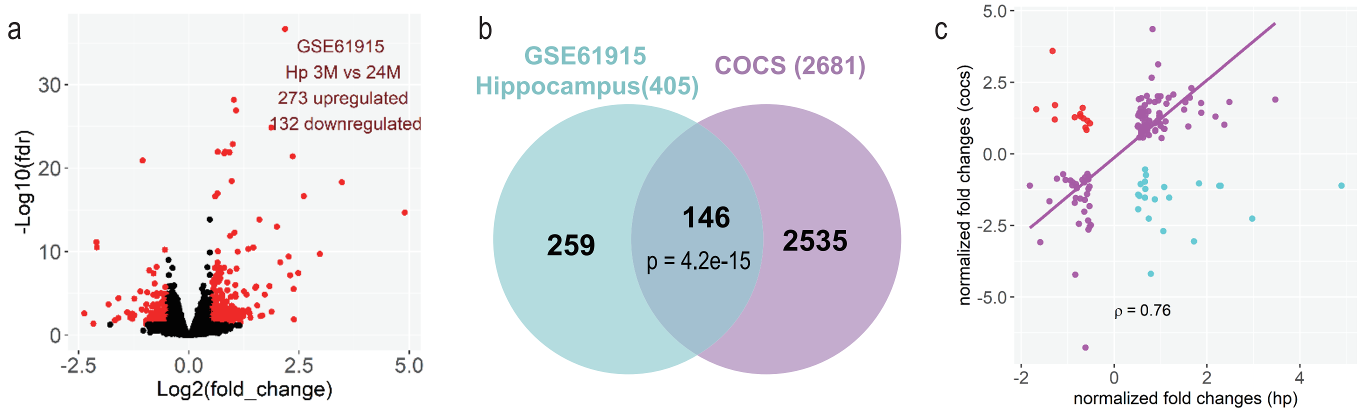

Supplementary figure 2, a, Volcano plot showing the dysregulated genes between the 24-month-old and 3-month-old mouse hippocampus (Hp). b, Venn diagram showing the overlaps between DEG in the aged COCS and the 24-month-old mouse Hp. p: hypergeometric test. c, Normalized fold changes of common DEG in aged COCS and the 24-month-old mouse Hp. ρ: Spearman's correlation coefficient. Genes show different directions of changes in the two conditions are highlighted in cyan or red.

Supplementary fig. 3, Liu et al.

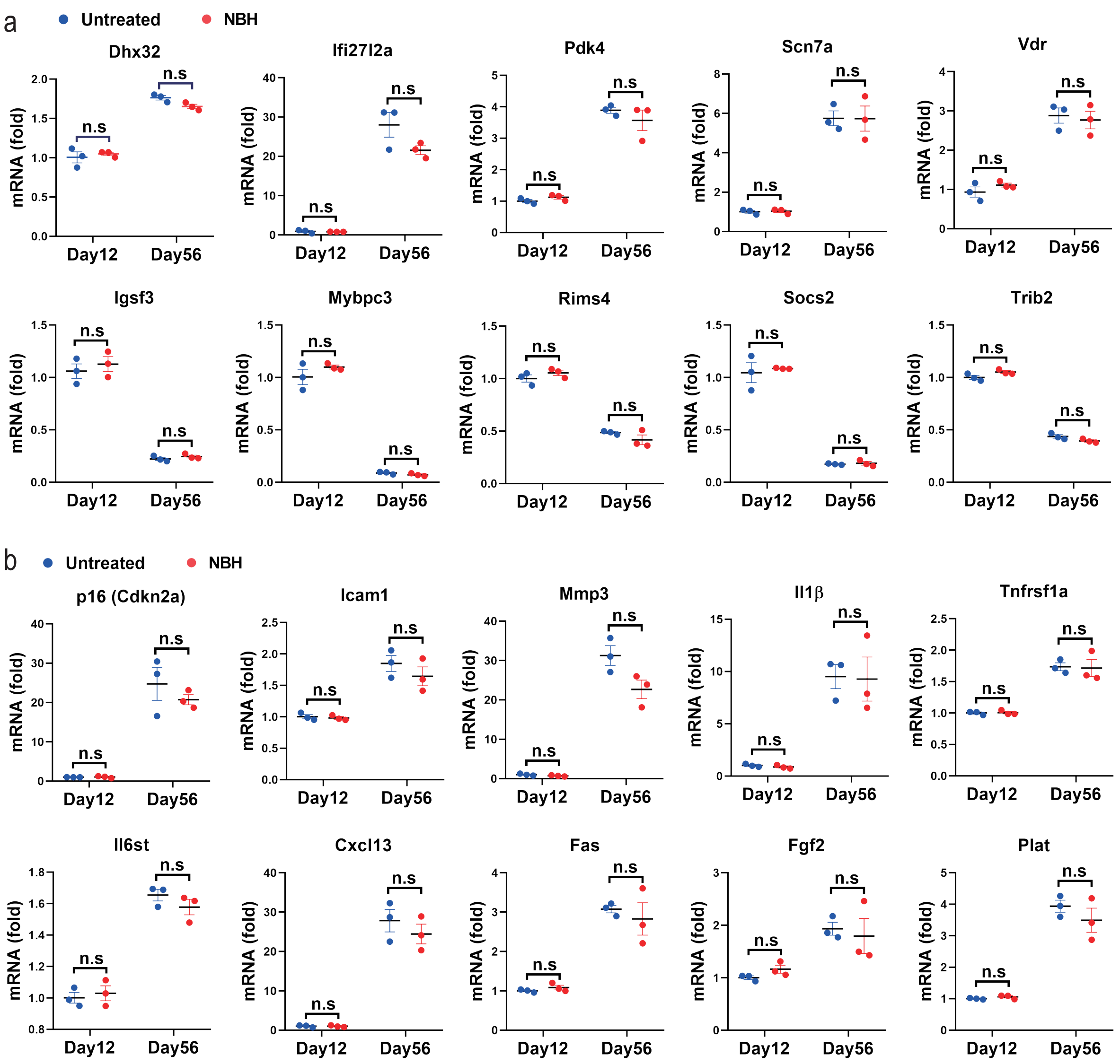

Supplementary figure 3, a, RT-PCR results showing similar expression levels of randomly picked age-predictive genes in naïve (untreated) and NBH-exposed COCS at day 12 and day 56 (n = 3). n.s: not significant. b, RT-PCR results showing similar expression levels of cellular senescence-associated genes in naïve (untreated) and NBH-exposed COCS at day 12 and day 56 (n = 3). n.s: not significant. Data are shown as mean +/- SEM.

Supplementary fig 4, Liu et al.

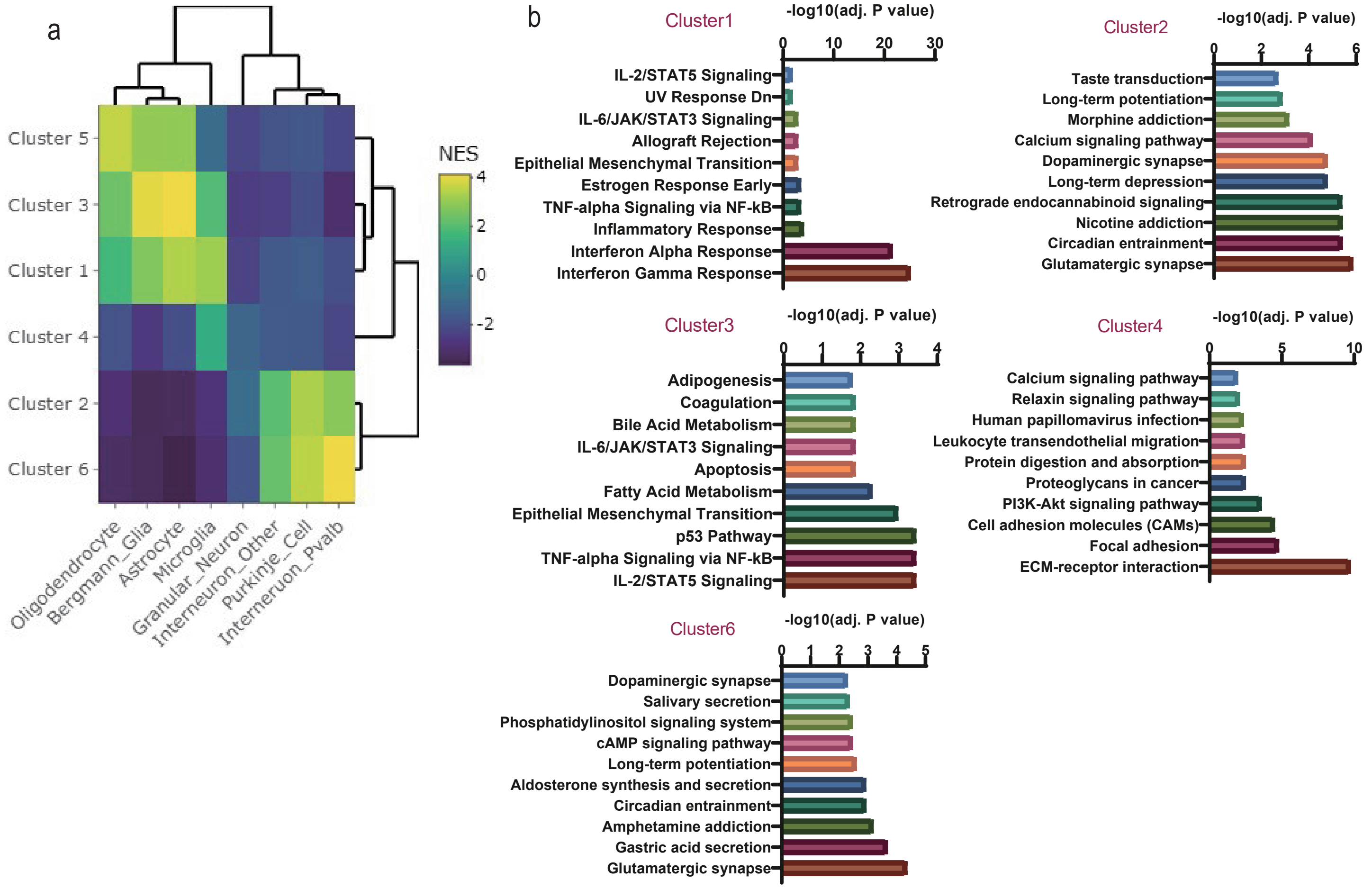

Supplementary figure 4, a, Heatmap showing the normalized enrichment score (NES) of gene set enrichment analysis of genes from the six clusters shown in fig. 3b for major cell types of the adult mouse cerebellum. b, Top 10 enriched pathways (adjusted p values < 0.05) for genes from the six clusters shown in fig. 3b. No pathways were significantly enriched for genes in cluster 5.

Supplementary fig 5, Liu et al.

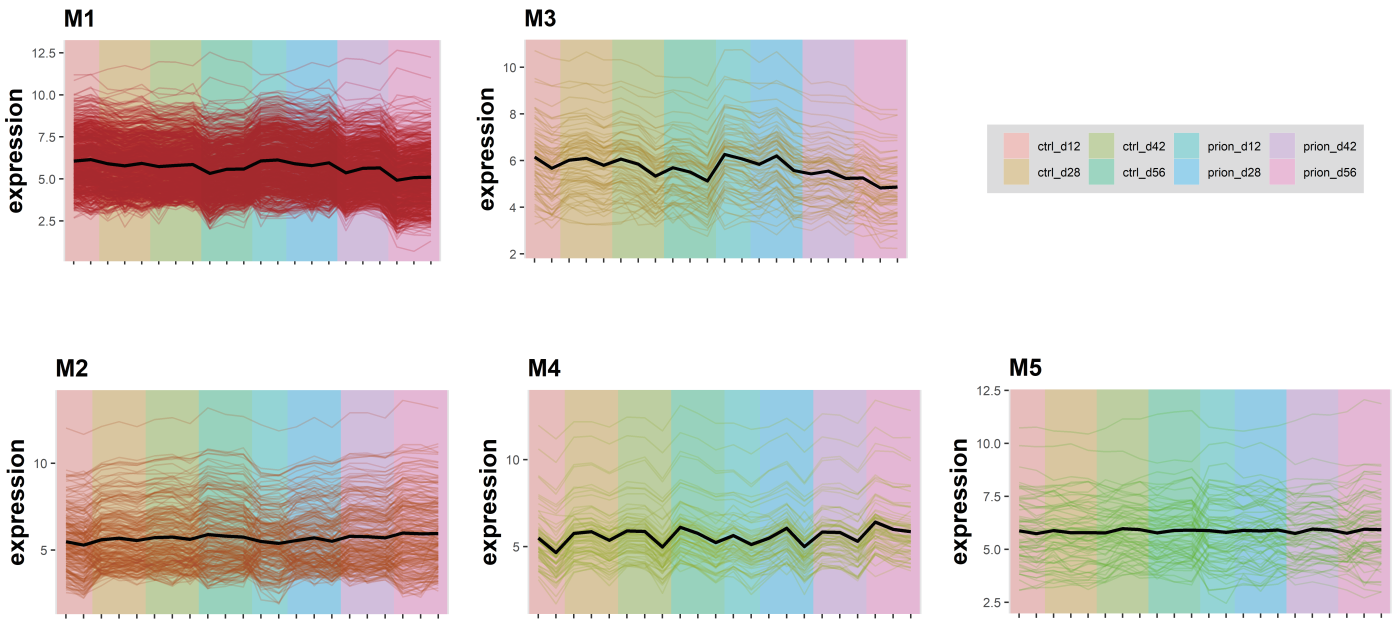

Supplementary figure 5, Temporal expression profiles of genes in the five gene co-expression network modules identified across experimental conditions.

Supplementary fig 6, Liu et al.

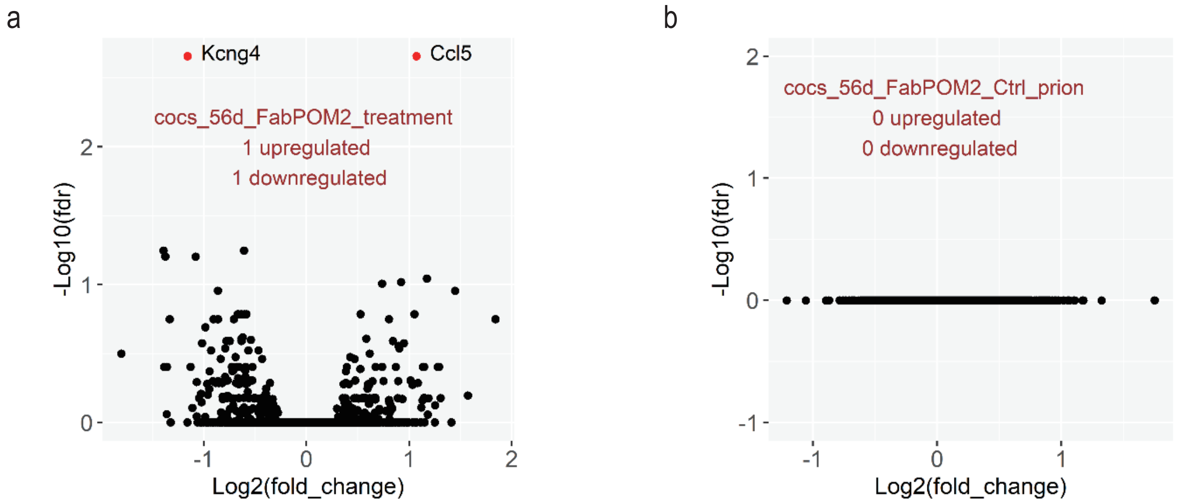

Supplementary figure 6, a, Volcano plot showing the only two differentially expressed genes (DEG) between FabPOM2 treated and untreated COCS at day 56 in the absence of prion infection. b, Volcano plot showing no DEG between the prion- and NBH (Ctrl) COCS treated with FabPOM2.

Supplementary fig 7, Liu et al.

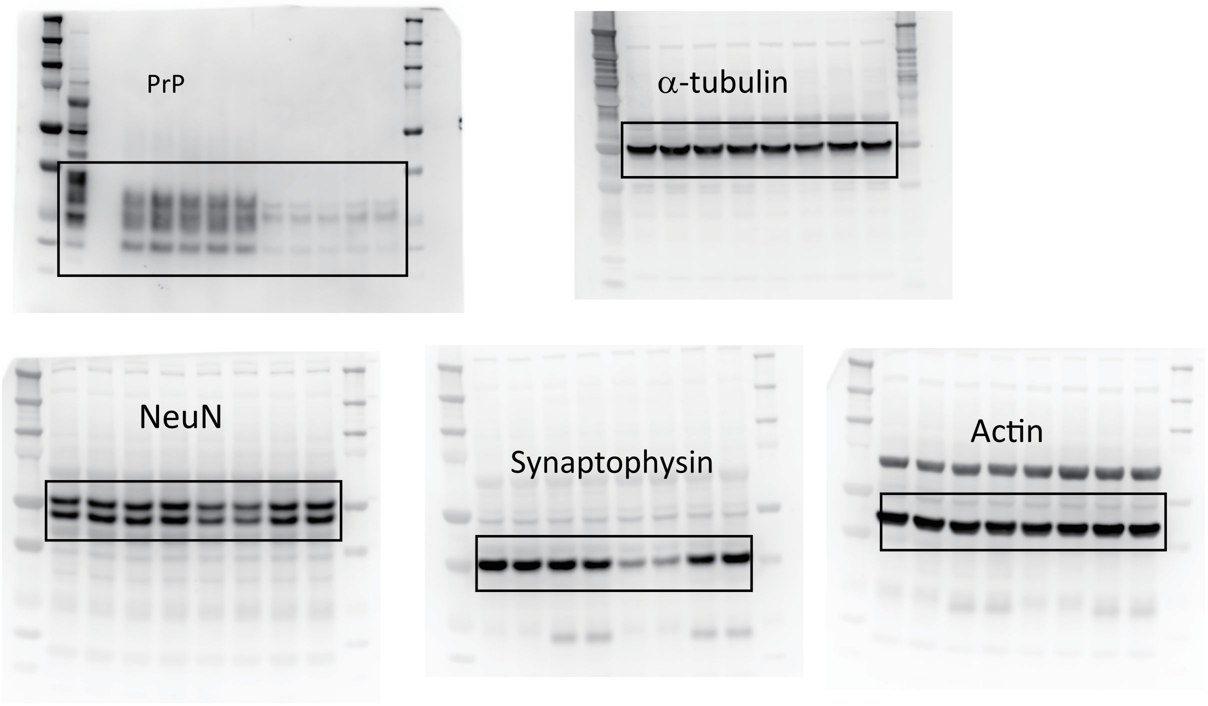

Supplementary figure 7, Raw images of western blots shown in Figure 4.
