# Supplementary material for: Brain aging is faithfully modelled in organotypic brain slices and accelerated by prions
Source: Commun Biol. 2022 Jun 8;5:557. doi: 10.1038/s42003-022-03496-5 (PMC9177860; doi:10.1038/s42003-022-03496-5)
Supplement: Supplementary file 2 — Description of Additional Supplementary Files [file 42003_2022_3496_MOESM2_ESM.pdf]

Description of Additional Supplementary Files

File name: Supplementary Data 1
Description: Differentially expressed genes in the control COCS between 56 and 12 days

File name: Supplementary Data 2
Description: Differentially expressed genes in the cerebellar tissues of NBH-inoculated mice between 56 and 182 days (~4-9 months of age)

File name: Supplementary Data 3
Description: Commonly altered KEGG pathways in aged COCS and in vivo

File name: Supplementary Data 4
Description: Age-predicting genes for organotypic cerebellar slices

File name: Supplementary Data 5
Description: The source data behind the graphs in the main figures
