# Supplementary material for: Brain aging is faithfully modelled in organotypic brain slices and accelerated by prions
Source: Commun Biol. 2022 Jun 8;5:557. doi: 10.1038/s42003-022-03496-5 (PMC9177860; doi:10.1038/s42003-022-03496-5)
Supplement: Supplementary file 8 — Reporting Summary [file 42003_2022_3496_MOESM8_ESM.pdf]

nature portfolio

Corresponding author(s): Yingjun Liu; Adriano Aguzzi

Last updated by author(s): Mar 30, 2022

Reporting Summary

Nature Portfolio wishes to improve the reproducibility of the work that we publish. This form provides structure for consistency and transparency in reporting. For further information on Nature Portfolio policies, see our Editorial Policies and the Editorial Policy Checklist.

Statistics

For all statistical analyses, confirm that the following items are present in the figure legend, table legend, main text, or Methods section.

n/a	Confirmed	
☐	☒	The exact sample size (*n*) for each experimental group/condition, given as a discrete number and unit of measurement
☐	☒	A statement on whether measurements were taken from distinct samples or whether the same sample was measured repeatedly
☐	☒	The statistical test(s) used AND whether they are one- or two-sided<br>*Only common tests should be described solely by name; describe more complex techniques in the Methods section.*
☐	☒	A description of all covariates tested
☐	☒	A description of any assumptions or corrections, such as tests of normality and adjustment for multiple comparisons
☐	☒	A full description of the statistical parameters including central tendency (e.g. means) or other basic estimates (e.g. regression coefficient) AND variation (e.g. standard deviation) or associated estimates of uncertainty (e.g. confidence intervals)
☐	☒	For null hypothesis testing, the test statistic (e.g. *F*, *t*, *r*) with confidence intervals, effect sizes, degrees of freedom and *P* value noted<br>*Give P values as exact values whenever suitable.*
☒	☐	For Bayesian analysis, information on the choice of priors and Markov chain Monte Carlo settings
☒	☐	For hierarchical and complex designs, identification of the appropriate level for tests and full reporting of outcomes
☒	☐	Estimates of effect sizes (e.g. Cohen's *d*, Pearson's *r*), indicating how they were calculated

Our web collection on statistics for biologists contains articles on many of the points above.

Software and code

Policy information about availability of computer code

Data collection	High throughput RNA-seq was performed on Illumina Novaseq 6000 using provided software. Membranes of western blots were digitized with ImageQuant (LAS-4000; Fujifilm) using provided software. Immunofluorescent staining was captured with a fluorescent microscope (Leica Biosystems) using provided software.
Data analysis	R version 4.0.3 was used for analyzing and visualizing the RNA-seq data. The following R packages were used: 'edgeR' version 3.30.3, 'tidyverse' version 1.3.0, 'GGally' version 2.1.1, 'ggpubr' version 0.4.0, 'ggrepel' version 0.8.2, 'pathfindR' version 1.6.0, 'psych' version 2.1.3, 'heatmaply' version 1.1.1, 'Mfuzz' version 2.48.0, 'marray' version 1.66.0, 'fgsea' version 1.14.0, 'CEMiTool' version 1.12.2. 'Caret' version 6.0-90. ImageJ 1.51f was used for quantify western blotting and immunofluorescent data. Graphpad prism 8.0 was used for visualizing and analyzing western blotting and immunofluorescent data.

For manuscripts utilizing custom algorithms or software that are central to the research but not yet described in published literature, software must be made available to editors and reviewers. We strongly encourage code deposition in a community repository (e.g. GitHub). See the Nature Portfolio guidelines for submitting code & software for further information.

March 2021

Data

Policy information about availability of data

All manuscripts must include a data availability statement. This statement should provide the following information, where applicable:

- Accession codes, unique identifiers, or web links for publicly available datasets
- A description of any restrictions on data availability
- For clinical datasets or third party data, please ensure that the statement adheres to our policy

Raw RNAseq data are available on ArrayExpress with the accession number E-MTAB-11635 and E-MTAB-11742. Raw images of western blots shown in the main figures are included in Supplementary Fig. 7. The source data for graphs in the main figures are included in Supplementary Data 5.

Field-specific reporting

Please select the one below that is the best fit for your research. If you are not sure, read the appropriate sections before making your selection.

☒ Life sciences ☐ Behavioural & social sciences ☐ Ecological, evolutionary & environmental sciences

For a reference copy of the document with all sections, see nature.com/documents/nr-reporting-summary-flat.pdf

Life sciences study design

All studies must disclose on these points even when the disclosure is negative.

Sample size	Sample size were based on literature and were not determined by statistical methods.
Data exclusions	No data were excluded from analysis.
Replication	The RNAseq experiments were performed with multiple biological replicates (different mice) per condition without technical replicates. All other experiments were performed with both biological and technical replicates, and all attempts at replication were successful.
Randomization	Mice were randomly assigned to experimental conditions and time points.
Blinding	The investigators were blinded for animal grouping, treatment, tissue and data collection.

Reporting for specific materials, systems and methods

We require information from authors about some types of materials, experimental systems and methods used in many studies. Here, indicate whether each material, system or method listed is relevant to your study. If you are not sure if a list item applies to your research, read the appropriate section before selecting a response.

Materials & experimental systems

n/a	Involved in the study	
☐	☒	Antibodies
☒	☐	Eukaryotic cell lines
☒	☐	Palaeontology and archaeology
☐	☒	Animals and other organisms
☒	☐	Human research participants
☒	☐	Clinical data
☒	☐	Dual use research of concern

Methods

n/a	Involved in the study	
☒	☐	ChIP-seq
☒	☐	Flow cytometry
☒	☐	MRI-based neuroimaging

Antibodies

Antibodies used	The following antibodies were used: mouse monoclonal antibodies against actin (Merck Millipore, MAB1501R), synaptophysin (BD Biosciences, BD611880) and PrP (POM1 and FabPOM2, home-made); rabbit monoclonal antibody against NeuN (Abcam, ab177487); rabbit polyclonal antibodies against alpha-tubulin (Proteintech, 11224-1-AP)
Validation	All antibodies used in the current study had been validated by the manufacturers and in previous publications.

Animals and other organisms

Policy information about studies involving animals; ARRIVE guidelines recommended for reporting animal research

Laboratory animals	Adult male C57BL/6J mice and postnatal day 10-12 C57BL/6J pups (both sexes) were used in the current study.
Wild animals	The study did not include any wild animals
Field-collected samples	The study did not include any samples collected at the field
Ethics oversight	All animal experiments were performed according to Swiss federal guidelines and approved by the Animal Experimentation Committee of the Canton of Zurich.

Note that full information on the approval of the study protocol must also be provided in the manuscript.
